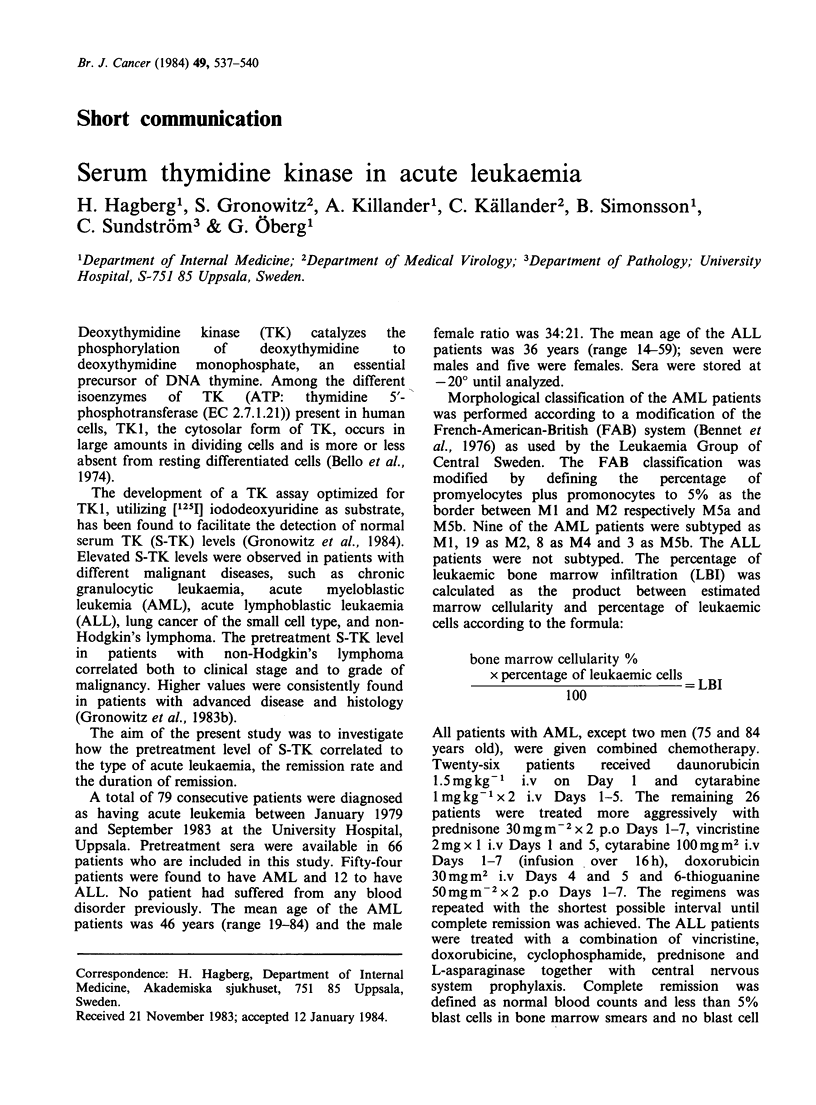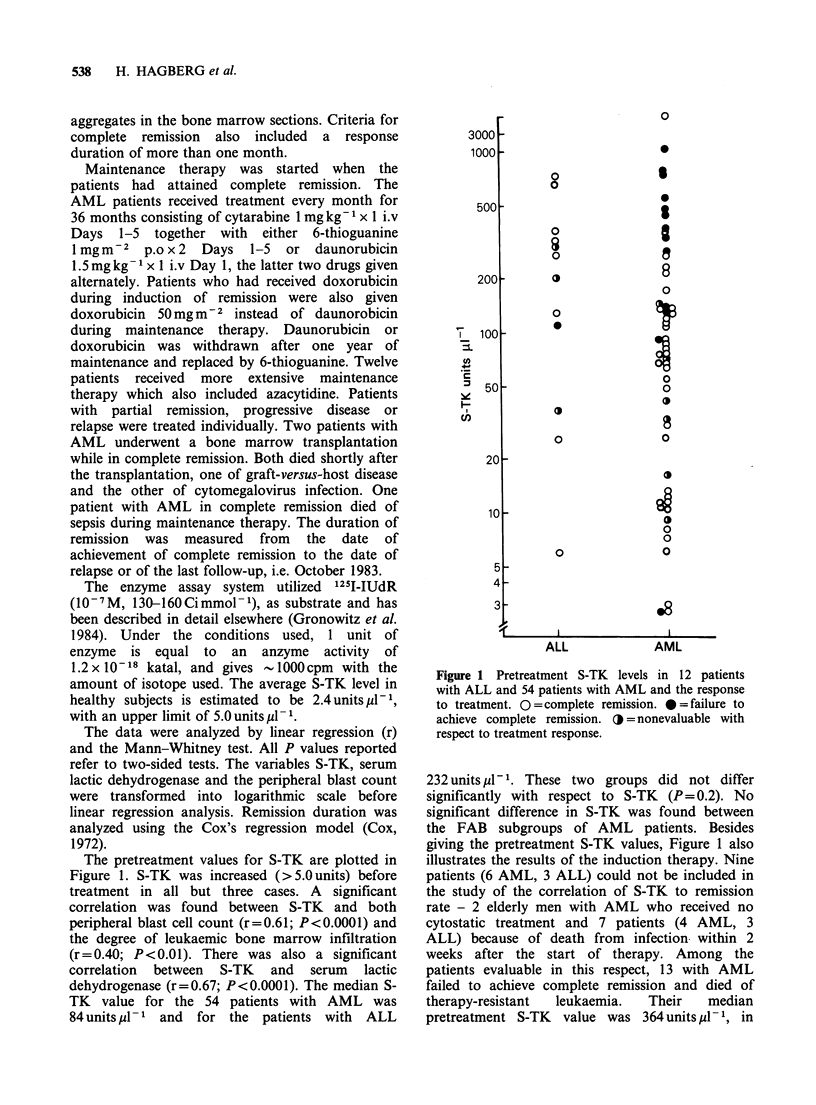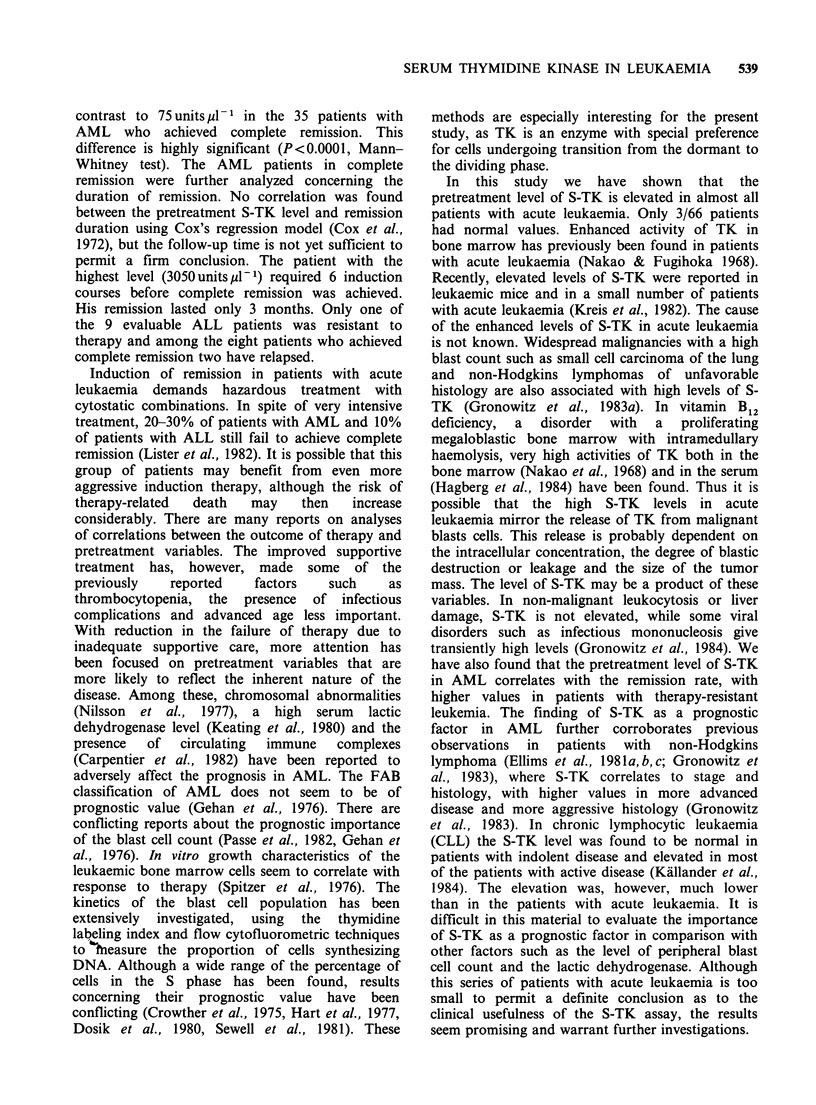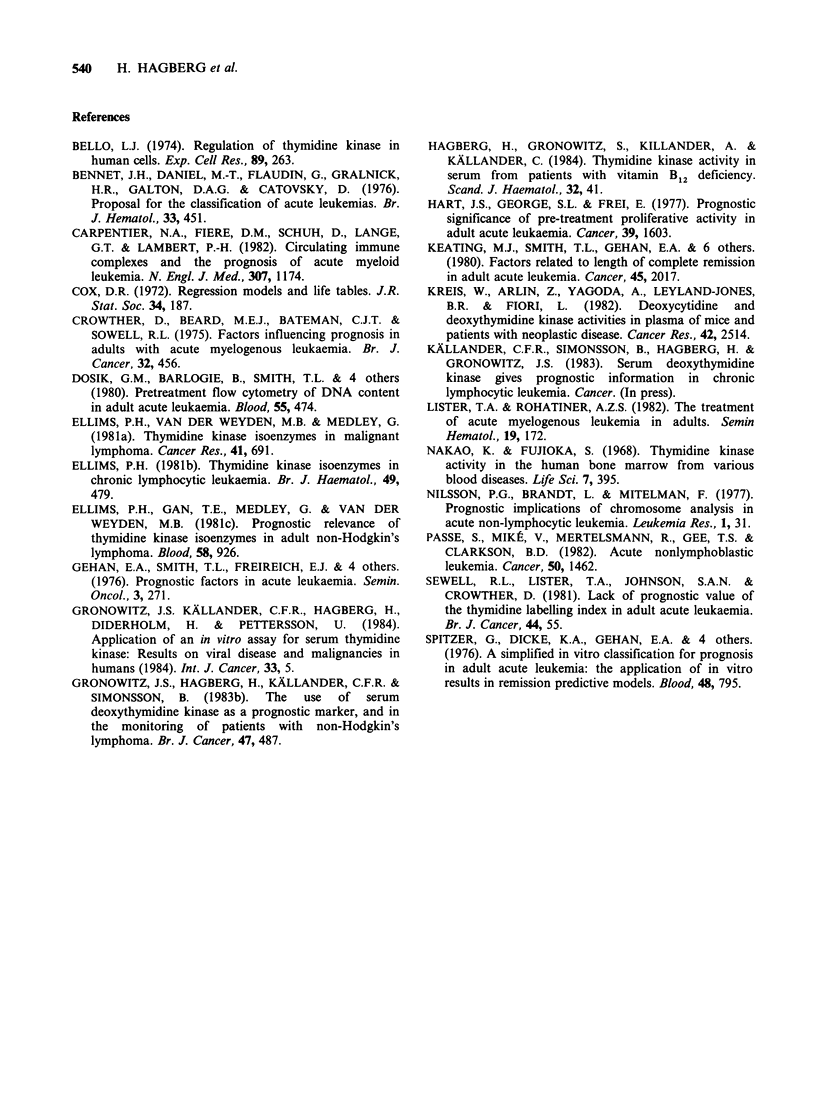# Serum thymidine kinase in acute leukaemia.

**DOI:** 10.1038/bjc.1984.82

**Published:** 1984-04

**Authors:** H. Hagberg, S. Gronowitz, A. Killander, C. Källander, B. Simonsson, C. Sundström, G. Oberg


Br. J. Cancer (1984) 49, 537–540

### Short communication

### Serum thymidine kinase in acute leukaemia

H. Hagberg[1], S. Gronowitz[2], A. Killander[1], C. Källander[2], B. Simonsson[1], C. Sundström[3] & G. Öberg[1]

[1]*Department of Internal Medicine;* [2]*Department of Medical Virology;* [3]*Department of Pathology; University Hospital, S-751 85 Uppsala, Sweden.*

Deoxythymidine kinase (TK) catalyzes the phosphorylation of deoxythymidine to deoxythymidine monophosphate, an essential precursor of DNA thymine. Among the different isoenzymes of TK (ATP: thymidine 5′-phosphotransferase (EC 2.7.1.21)) present in human cells, TK1, the cytosolar form of TK, occurs in large amounts in dividing cells and is more or less absent from resting differentiated cells (Bello *et al.*, 1974).

The development of a TK assay optimized for TK1, utilizing [$^{125}$I] iododeoxyuridine as substrate, has been found to facilitate the detection of normal serum TK (S-TK) levels (Gronowitz *et al.*, 1984). Elevated S-TK levels were observed in patients with different malignant diseases, such as chronic granulocytic leukaemia, acute myeloblastic leukemia (AML), acute lymphoblastic leukaemia (ALL), lung cancer of the small cell type, and non-Hodgkin's lymphoma. The pretreatment S-TK level in patients with non-Hodgkin's lymphoma correlated both to clinical stage and to grade of malignancy. Higher values were consistently found in patients with advanced disease and histology (Gronowitz *et al.*, 1983b).

The aim of the present study was to investigate how the pretreatment level of S-TK correlated to the type of acute leukaemia, the remission rate and the duration of remission.

A total of 79 consecutive patients were diagnosed as having acute leukemia between January 1979 and September 1983 at the University Hospital, Uppsala. Pretreatment sera were available in 66 patients who are included in this study. Fifty-four patients were found to have AML and 12 to have ALL. No patient had suffered from any blood disorder previously. The mean age of the AML patients was 46 years (range 19–84) and the male female ratio was 34:21. The mean age of the ALL patients was 36 years (range 14–59); seven were males and five were females. Sera were stored at −20° until analyzed.

Morphological classification of the AML patients was performed according to a modification of the French-American-British (FAB) system (Bennet *et al.*, 1976) as used by the Leukaemia Group of Central Sweden. The FAB classification was modified by defining the percentage of promyelocytes plus promonocytes to 5% as the border between M1 and M2 respectively M5a and M5b. Nine of the AML patients were subtyped as M1, 19 as M2, 8 as M4 and 3 as M5b. The ALL patients were not subtyped. The percentage of leukaemic bone marrow infiltration (LBI) was calculated as the product between estimated marrow cellularity and percentage of leukaemic cells according to the formula:

$$\frac{\text{bone marrow cellularity \%} \times \text{percentage of leukaemic cells}}{100} = \text{LBI}$$

All patients with AML, except two men (75 and 84 years old), were given combined chemotherapy. Twenty-six patients received daunorubicin $1.5\,\text{mg}\,\text{kg}^{-1}$ i.v on Day 1 and cytarabine $1\,\text{mg}\,\text{kg}^{-1} \times 2$ i.v Days 1–5. The remaining 26 patients were treated more aggressively with prednisone $30\,\text{mg}\,\text{m}^{-2} \times 2$ p.o Days 1–7, vincristine $2\,\text{mg} \times 1$ i.v Days 1 and 5, cytarabine $100\,\text{mg}\,\text{m}^2$ i.v Days 1–7 (infusion over 16 h), doxorubicin $30\,\text{mg}\,\text{m}^2$ i.v Days 4 and 5 and 6-thioguanine $50\,\text{mg}\,\text{m}^{-2} \times 2$ p.o Days 1–7. The regimens was repeated with the shortest possible interval until complete remission was achieved. The ALL patients were treated with a combination of vincristine, doxorubicine, cyclophosphamide, prednisone and L-asparaginase together with central nervous system prophylaxis. Complete remission was defined as normal blood counts and less than 5% blast cells in bone marrow smears and no blast cell

Correspondence: H. Hagberg, Department of Internal Medicine, Akademiska sjukhuset, 751 85 Uppsala, Sweden.
Received 21 November 1983; accepted 12 January 1984.

aggregates in the bone marrow sections. Criteria for complete remission also included a response duration of more than one month.

Maintenance therapy was started when the patients had attained complete remission. The AML patients received treatment every month for 36 months consisting of cytarabine $1\,mg\,kg^{-1} \times 1$ i.v Days 1–5 together with either 6-thioguanine $1\,mg\,m^{-2}$ p.o $\times 2$ Days 1–5 or daunorubicin $1.5\,mg\,kg^{-1} \times 1$ i.v Day 1, the latter two drugs given alternately. Patients who had received doxorubicin during induction of remission were also given doxorubicin $50\,mg\,m^{-2}$ instead of daunorobicin during maintenance therapy. Daunorubicin or doxorubicin was withdrawn after one year of maintenance and replaced by 6-thioguanine. Twelve patients received more extensive maintenance therapy which also included azacytidine. Patients with partial remission, progressive disease or relapse were treated individually. Two patients with AML underwent a bone marrow transplantation while in complete remission. Both died shortly after the transplantation, one of graft-*versus*-host disease and the other of cytomegalovirus infection. One patient with AML in complete remission died of sepsis during maintenance therapy. The duration of remission was measured from the date of achievement of complete remission to the date of relapse or of the last follow-up, i.e. October 1983.

The enzyme assay system utilized $^{125}$I-IUdR ($10^{-7}$M, $130–160\,Ci\,mmol^{-1}$), as substrate and has been described in detail elsewhere (Gronowitz *et al.* 1984). Under the conditions used, 1 unit of enzyme is equal to an anzyme activity of $1.2 \times 10^{-18}$ katal, and gives $\sim 1000$ cpm with the amount of isotope used. The average S-TK level in healthy subjects is estimated to be $2.4\,units\,\mu l^{-1}$, with an upper limit of $5.0\,units\,\mu l^{-1}$.

The data were analyzed by linear regression (r) and the Mann–Whitney test. All $P$ values reported refer to two-sided tests. The variables S-TK, serum lactic dehydrogenase and the peripheral blast count were transformed into logarithmic scale before linear regression analysis. Remission duration was analyzed using the Cox's regression model (Cox, 1972).

The pretreatment values for S-TK are plotted in Figure 1. S-TK was increased ($>5.0$ units) before treatment in all but three cases. A significant correlation was found between S-TK and both peripheral blast cell count ($r=0.61$; $P<0.0001$) and the degree of leukaemic bone marrow infiltration ($r=0.40$; $P<0.01$). There was also a significant correlation between S-TK and serum lactic dehydrogenase ($r=0.67$; $P<0.0001$). The median S-TK value for the 54 patients with AML was $84\,units\,\mu l^{-1}$ and for the patients with ALL

**Figure 1** Pretreatment S-TK levels in 12 patients with ALL and 54 patients with AML and the response to treatment. ○ = complete remission. ● = failure to achieve complete remission. ◑ = nonevaluable with respect to treatment response.

$232\,units\,\mu l^{-1}$. These two groups did not differ significantly with respect to S-TK ($P=0.2$). No significant difference in S-TK was found between the FAB subgroups of AML patients. Besides giving the pretreatment S-TK values, Figure 1 also illustrates the results of the induction therapy. Nine patients (6 AML, 3 ALL) could not be included in the study of the correlation of S-TK to remission rate – 2 elderly men with AML who received no cytostatic treatment and 7 patients (4 AML, 3 ALL) because of death from infection within 2 weeks after the start of therapy. Among the patients evaluable in this respect, 13 with AML failed to achieve complete remission and died of therapy-resistant leukaemia. Their median pretreatment S-TK value was $364\,units\,\mu l^{-1}$, in contrast to 75 units $\mu l^{-1}$ in the 35 patients with AML who achieved complete remission. This difference is highly significant ($P<0.0001$, Mann–Whitney test). The AML patients in complete remission were further analyzed concerning the duration of remission. No correlation was found between the pretreatment S-TK level and remission duration using Cox's regression model (Cox *et al.*, 1972), but the follow-up time is not yet sufficient to permit a firm conclusion. The patient with the highest level (3050 units $\mu l^{-1}$) required 6 induction courses before complete remission was achieved. His remission lasted only 3 months. Only one of the 9 evaluable ALL patients was resistant to therapy and among the eight patients who achieved complete remission two have relapsed.

Induction of remission in patients with acute leukaemia demands hazardous treatment with cytostatic combinations. In spite of very intensive treatment, 20–30% of patients with AML and 10% of patients with ALL still fail to achieve complete remission (Lister *et al.*, 1982). It is possible that this group of patients may benefit from even more aggressive induction therapy, although the risk of therapy-related death may then increase considerably. There are many reports on analyses of correlations between the outcome of therapy and pretreatment variables. The improved supportive treatment has, however, made some of the previously reported factors such as thrombocytopenia, the presence of infectious complications and advanced age less important. With reduction in the failure of therapy due to inadequate supportive care, more attention has been focused on pretreatment variables that are more likely to reflect the inherent nature of the disease. Among these, chromosomal abnormalities (Nilsson *et al.*, 1977), a high serum lactic dehydrogenase level (Keating *et al.*, 1980) and the presence of circulating immune complexes (Carpentier *et al.*, 1982) have been reported to adversely affect the prognosis in AML. The FAB classification of AML does not seem to be of prognostic value (Gehan *et al.*, 1976). There are conflicting reports about the prognostic importance of the blast cell count (Passe *et al.*, 1982, Gehan *et al.*, 1976). *In vitro* growth characteristics of the leukaemic bone marrow cells seem to correlate with response to therapy (Spitzer *et al.*, 1976). The kinetics of the blast cell population has been extensively investigated, using the thymidine labeling index and flow cytofluorometric techniques to measure the proportion of cells synthesizing DNA. Although a wide range of the percentage of cells in the S phase has been found, results concerning their prognostic value have been conflicting (Crowther *et al.*, 1975, Hart *et al.*, 1977, Dosik *et al.*, 1980, Sewell *et al.*, 1981). These methods are especially interesting for the present study, as TK is an enzyme with special preference for cells undergoing transition from the dormant to the dividing phase.

In this study we have shown that the pretreatment level of S-TK is elevated in almost all patients with acute leukaemia. Only 3/66 patients had normal values. Enhanced activity of TK in bone marrow has previously been found in patients with acute leukaemia (Nakao & Fugihoka 1968). Recently, elevated levels of S-TK were reported in leukaemic mice and in a small number of patients with acute leukaemia (Kreis *et al.*, 1982). The cause of the enhanced levels of S-TK in acute leukaemia is not known. Widespread malignancies with a high blast count such as small cell carcinoma of the lung and non-Hodgkins lymphomas of unfavorable histology are also associated with high levels of S-TK (Gronowitz *et al.*, 1983*a*). In vitamin $B_{12}$ deficiency, a disorder with a proliferating megaloblastic bone marrow with intramedullary haemolysis, very high activities of TK both in the bone marrow (Nakao *et al.*, 1968) and in the serum (Hagberg *et al.*, 1984) have been found. Thus it is possible that the high S-TK levels in acute leukaemia mirror the release of TK from malignant blasts cells. This release is probably dependent on the intracellular concentration, the degree of blastic destruction or leakage and the size of the tumor mass. The level of S-TK may be a product of these variables. In non-malignant leukocytosis or liver damage, S-TK is not elevated, while some viral disorders such as infectious mononucleosis give transiently high levels (Gronowitz *et al.*, 1984). We have also found that the pretreatment level of S-TK in AML correlates with the remission rate, with higher values in patients with therapy-resistant leukemia. The finding of S-TK as a prognostic factor in AML further corroborates previous observations in patients with non-Hodgkins lymphoma (Ellims *et al.*, 1981*a,b,c*; Gronowitz *et al.*, 1983), where S-TK correlates to stage and histology, with higher values in more advanced disease and more aggressive histology (Gronowitz *et al.*, 1983). In chronic lymphocytic leukaemia (CLL) the S-TK level was found to be normal in patients with indolent disease and elevated in most of the patients with active disease (Källander *et al.*, 1984). The elevation was, however, much lower than in the patients with acute leukaemia. It is difficult in this material to evaluate the importance of S-TK as a prognostic factor in comparison with other factors such as the level of peripheral blast cell count and the lactic dehydrogenase. Although this series of patients with acute leukaemia is too small to permit a definite conclusion as to the clinical usefulness of the S-TK assay, the results seem promising and warrant further investigations.

### References

BELLO, L.J. (1974). Regulation of thymidine kinase in human cells. *Exp. Cell Res.*, **89**, 263.

BENNET, J.H., DANIEL, M.-T., FLAUDIN, G., GRALNICK, H.R., GALTON, D.A.G. & CATOVSKY, D. (1976). Proposal for the classification of acute leukemias. *Br. J. Hematol.*, **33**, 451.

CARPENTIER, N.A., FIERE, D.M., SCHUH, D., LANGE, G.T. & LAMBERT, P.-H. (1982). Circulating immune complexes and the prognosis of acute myeloid leukemia. *N. Engl. J. Med.*, **307**, 1174.

COX, D.R. (1972). Regression models and life tables. *J.R. Stat. Soc.* **34**, 187.

CROWTHER, D., BEARD, M.E.J., BATEMAN, C.J.T. & SOWELL, R.L. (1975). Factors influencing prognosis in adults with acute myelogenous leukaemia. *Br. J. Cancer*, **32**, 456.

DOSIK, G.M., BARLOGIE, B., SMITH, T.L. & 4 others (1980). Pretreatment flow cytometry of DNA content in adult acute leukaemia. *Blood*, **55**, 474.

ELLIMS, P.H., VAN DER WEYDEN, M.B. & MEDLEY, G. (1981a). Thymidine kinase isoenzymes in malignant lymphoma. *Cancer Res.*, **41**, 691.

ELLIMS, P.H. (1981b). Thymidine kinase isoenzymes in chronic lymphocytic leukaemia. *Br. J. Haematol.*, **49**, 479.

ELLIMS, P.H., GAN, T.E., MEDLEY, G. & VAN DER WEYDEN, M.B. (1981c). Prognostic relevance of thymidine kinase isoenzymes in adult non-Hodgkin's lymphoma. *Blood*, **58**, 926.

GEHAN, E.A., SMITH, T.L., FREIREICH, E.J. & 4 others. (1976). Prognostic factors in acute leukaemia. *Semin. Oncol.*, **3**, 271.

GRONOWITZ, J.S. KÄLLANDER, C.F.R., HAGBERG, H., DIDERHOLM, H. & PETTERSSON, U. (1984). Application of an *in vitro* assay for serum thymidine kinase: Results on viral disease and malignancies in humans (1984). *Int. J. Cancer*, **33**, 5.

GRONOWITZ, J.S., HAGBERG, H., KÄLLANDER, C.F.R. & SIMONSSON, B. (1983b). The use of serum deoxythymidine kinase as a prognostic marker, and in the monitoring of patients with non-Hodgkin's lymphoma. *Br. J. Cancer*, **47**, 487.

HAGBERG, H., GRONOWITZ, S., KILLANDER, A. & KÄLLANDER, C. (1984). Thymidine kinase activity in serum from patients with vitamin $B_{12}$ deficiency. *Scand. J. Haematol.*, **32**, 41.

HART, J.S., GEORGE, S.L. & FREI, E. (1977). Prognostic significance of pre-treatment proliferative activity in adult acute leukaemia. *Cancer*, **39**, 1603.

KEATING, M.J., SMITH, T.L., GEHAN, E.A. & 6 others. (1980). Factors related to length of complete remission in adult acute leukemia. *Cancer*, **45**, 2017.

KREIS, W., ARLIN, Z., YAGODA, A., LEYLAND-JONES, B.R. & FIORI, L. (1982). Deoxycytidine and deoxythymidine kinase activities in plasma of mice and patients with neoplastic disease. *Cancer Res.*, **42**, 2514.

KÄLLANDER, C.F.R., SIMONSSON, B., HAGBERG, H. & GRONOWITZ, J.S. (1983). Serum deoxythymidine kinase gives prognostic information in chronic lymphocytic leukemia. *Cancer*. (In press).

LISTER, T.A. & ROHATINER, A.Z.S. (1982). The treatment of acute myelogenous leukemia in adults. *Semin Hematol.*, **19**, 172.

NAKAO, K. & FUJIOKA, S. (1968). Thymidine kinase activity in the human bone marrow from various blood diseases. *Life Sci.* **7**, 395.

NILSSON, P.G., BRANDT, L. & MITELMAN, F. (1977). Prognostic implications of chromosome analysis in acute non-lymphocytic leukemia. *Leukemia Res.*, **1**, 31.

PASSE, S., MIKÉ, V., MERTELSMANN, R., GEE, T.S. & CLARKSON, B.D. (1982). Acute nonlymphoblastic leukemia. *Cancer*, **50**, 1462.

SEWELL, R.L., LISTER, T.A., JOHNSON, S.A.N. & CROWTHER, D. (1981). Lack of prognostic value of the thymidine labelling index in adult acute leukaemia. *Br. J. Cancer*, **44**, 55.

SPITZER, G., DICKE, K.A., GEHAN, E.A. & 4 others. (1976). A simplified in vitro classification for prognosis in adult acute leukemia: the application of in vitro results in remission predictive models. *Blood*, **48**, 795.